# The K_Ca_2 Channel Inhibitor AP14145, But Not Dofetilide or Ondansetron, Provides Functional Atrial Selectivity in Guinea Pig Hearts

**DOI:** 10.3389/fphar.2019.00668

**Published:** 2019-06-19

**Authors:** Jeppe Egedal Kirchhoff, Mark Alexander Skarsfeldt, Kalai Mangai Muthukumarasamy, Rafel Simó-Vicens, Sofia Hammami Bomholtz, Lea Abildgaard, Thomas Jespersen, Ulrik S. Sørensen, Morten Grunnet, Bo Hjorth Bentzen, Jonas Goldin Diness

**Affiliations:** ^1^Acesion Pharma, Copenhagen, Denmark; ^2^Department of Biomedical Sciences, Faculty of Health and Medical Sciences, University of Copenhagen, Copenhagen, Denmark

**Keywords:** arrhythmia (heart rhythm disorders), dofetilide, ondansetron, AP14145, QT prolongation

## Abstract

**Background and Purpose:** Prolongation of cardiac action potentials is considered antiarrhythmic in the atria but can be proarrhythmic in ventricles if the current carried by Kv11.1-channels (I_Kr_) is inhibited. The current mediated by K_Ca_2-channels, I_KCa_, is considered a promising new target for treatment of atrial fibrillation (AF). Selective inhibitors of I_Kr_ (dofetilide) and I_KCa_ (AP14145) were used to compare the effects on ventricular and atrial repolarization. Ondansetron, which has been reported to be a potent blocker of both I_Kr_ and I_KCa_, was included to examine its potential atrial antiarrhythmic properties.

**Experimental Approach:** The expression of K_Ca_2- and K_v_11.1-channels in the guinea pig heart was investigated using quantitative polymerase chain reaction (qPCR). Whole-cell patch clamp technique was used to investigate the effects of dofetilide, AP14145, and ondansetron on I_KCa_ and/or I_Kr_. The effect of dofetilide, AP14145, and ondansetron on atrial and ventricular repolarization was investigated in isolated hearts. A novel atrial paced *in vivo* guinea pig model was further validated using AP14145 and dofetilide.

**Key Results:** AP14145 increased the atrial effective refractory period (AERP) without prolonging the QT interval with Bazett’s correction for heart rate (QTcB) both *ex vivo* and *in vivo*. In contrast, dofetilide increased QTcB and, to a lesser extent, AERP in isolated hearts and prolonged QTcB with no effects on AERP in the *in vivo* guinea pig model. Ondansetron did not inhibit I_KCa_, but did inhibit I_Kr_ *in vitro*. Ondansetron prolonged ventricular, but not atrial repolarization *ex vivo*.

**Conclusion and Implications:** I_KCa_ inhibition by AP14145 selectively increases atrial repolarization, whereas I_Kr_ inhibition by dofetilide and ondansetron increases ventricular repolarization to a larger extent than atrial repolarization.

## Introduction

Atrial fibrillation (AF) is the most prevalent sustained cardiac arrhythmia and is associated with reduced quality of life and increased mortality and morbidity (Heeringa et al., 2006; Piccini et al., 2012). AF is often treated with antiarrhythmic drugs that affect the electrical properties of ion channels in the heart (Zimetbaum, 2012). Class III antiarrhythmic compounds exert their effects by decreasing cardiac K^+^ currents leading to delayed repolarization and a concomitant increased effective refractory period. Prolonging the atrial effective refractory period (AERP) is antiarrhythmic, whereas drug induced prolongation of the ventricular repolarization that manifests as prolongation of the QT interval on a surface ECG is a risk marker for potentially lethal ventricular arrhythmias such as *torsades de pointes* for a wide range of drugs. For the majority of these compounds the QT prolongation is caused by inhibition of the rapidly activating delayed rectifier potassium current I_Kr_ carried by the K_v_11.1 (also known as the hERG) channel (Redfern et al., 2003). The QT interval corrected for heart rate (HR) using Bazett’s formula (QTcB) is therefore used as a surrogate marker for proarrhythmicity, albeit an imperfect one since not all drugs that prolong the QTcB are proarrhythmic (Piccini et al., 2009).

Dofetilide is an archetypical class III antiarrhythmic that selectively blocks I_Kr_ (Roukoz and Saliba, 2007). Although fairly effective at treating AF, the use of classical class III antiarrhythmic compounds has been limited by the risk of inducing potentially lethal ventricular arrhythmias (Waldo et al., 1996). During the last decades, substantial antiarrhythmic effects in normal and remodeled atria, without adverse effects in the ventricles, have therefore been a sought-after therapeutic goal (Grunnet et al., 2012).

AP14145 inhibits the K_Ca_2 channels, also known as small conductance Ca^2+^-activated K^+^, or SK, channels (Diness et al., 2017; Simó-Vicens et al., 2017a). The current mediated by this channel (I_KCa_) has emerged as a promising new target for AF treatment, because inhibition of this current can apparently prolong atrial repolarization without affecting the ventricular repolarization, thereby limiting the risk of ventricular adverse effects (Xu et al., 2003; Diness et al., 2010; Diness et al., 2017; Qi et al., 2013). Ondansetron is an antagonist of 5-HT_3_ receptors in the CNS and is used as an antiemetic. It has been reported to block both I_Kr_ and I_KCa_ at nanomolar concentrations (Kuryshev et al., 2000; Ko et al., 2018). Three subtypes of K_Ca_2 channels, K_Ca_2.1, K_Ca_2.2, and K_Ca_2.3, carry the I_KCa_ current. The K_Ca_2.2 and K_Ca_2.3 subtypes are predominantly expressed in the human atria (Skibsbye et al., 2014) and have been directly linked to human AF (Ellinor et al., 2010; Ellinor et al., 2012; Christophersen et al., 2017).

In this study, we directly compare the effects on surrogate markers for pro- and antiarrhythmicity, prolongation of QTcB and AERP of a classical, and an atrial selective class III antiarrhythmic; dofetilide and AP14145. We also test the effects of ondansetron on these parameters in order to investigate whether ondansetron can act as an atrial selective class III antiarrhythmic.

In order to investigate the effects of dofetilide, ondansetron, and AP14145 on both AERP and QTcB we tested the compounds in Langendorff perfused guinea pig hearts.

A novel closed chest *in vivo* guinea pig model was also developed. In this model a pacing catheter was placed in the right atrium, making it possible to investigate both AERP and QTcB as surrogate markers for anti- and proarrhythmicity of new chemical entities in a small animal *in vivo* model. Both AP14145 and dofetilide were tested in this model to examine the translatability of the Langendorff heart to an *in vivo* setting.

## Materials and Methods

This study was carried out in accordance with the recommendations of Danish guidelines for animal experiments according to the European Commission Directive 86/609/EEC, Danish Ministry of Justice. The protocol was approved by the Danish Ministry of Justice (license no. 2016-15-0201-00850).

### Drugs and Solutions

AP14145 was supplied by Acesion Pharma. Dofetilide was purchased at Sigma-Aldrich (St. Louis, USA) for the Langendorff and Hello Bio (Bristol, UK) for the closed chest *in vivo* experiments. Ondansetron was purchased at Sigma-Aldrich (St. Louis, USA). For the Langendorff perfused heart experiments the compounds were dissolved in DMSO to make stock solutions and diluted to the final concentration in the Krebs-Henseleit buffer during the experiments. The final concentration of DMSO never exceeded 0.3% in the buffer. For the closed chest experiments, the compounds were first dissolved in PEG-400 and then diluted to a 50% saline and 50% PEG-400 solution.

For patch clamp experiments, dofetilide, ondansetron, and AP14145 were solubilized in pure DMSO (Sigma-Aldrich, Germany) at 10 mM and bicuculline methiodide (Sigma-Aldrich, Germany) at 100-mM stock solutions. These stock solutions were stored at −20°C and aliquots were solubilized at the desired concentration on the day of the experiment.

The K_Ca_2.2 manual patch-clamp experiments and K_Ca_2.2/K_Ca_2.3 QPatch experiments were conducted using symmetrical K^+^ solutions. The extracellular solution contained (in mM) 0.1 CaCl_2_, 3 MgCl_2_, 154 KCl, 10 HEPES, and 10 glucose (pH = 7.4 and 285–295 mOsm). The intracellular solution contained (in mM) 8.106 CaCl_2_ (final free Ca^2+^ concentration of 400 nM), 1.167 MgCl_2_, 10 EGTA, 108 KCl, 10 HEPES, 31.25/10 KOH/EGTA, and 15 KOH (pH = 7.2).

The effect of ondansetron on I_KCa_ was studied by adding increasing concentration of drug (in µM: 0.1, 0.3, 1, 3, 10, and 30). Bicuculline methiodide was used as a positive control for K_Ca_2 specific current at the beginning and the end of the experiment.

For the K_v_11.1 experiments conducted on the automated QPatch machine, the extracellular solution contained (in mM) 145 NaCl, 4 KCl, 2 CaCl_2_, 1 MgCl_2_, 10 HEPES, and 10 glucose (pH = 7.4 and 305 mOsm adjusted with sucrose). The intracellular solution contained (in mM) 120 KCl, 31.25/10 KOH/EGTA, 5.4 CaCl_2_, 1.75 MgCl_2_, 4 mM Na_2_ATP, and 10 HEPES (pH = 7.2 and 285–296 mOsm adjusted with sucrose). Recordings were made before and after application of drug. To establish a half maximal inhibitory concentration (IC_50_) value, increasing concentration of drugs were applied: Dofetilide (in nM: 10, 25, 50, 100, 250, and 500), AP14145 (in µM: 1, 3, 10, 30, and 100), and ondansetron (in µM: 3, 10, 30, 60, 100, and 300). At the end of the experiment, 500 nM dofetilide was added as a reference drug, and the obtained current level during application of dofetilide was used as the maximal inhibitory response.

### *In Vitro* Electrophysiology

#### Cell Culture and Cell Preparation for Manual Patch Clamp

To study the effect of ondansetron on I_KCa_, wild-type HEK293 cells were transiently co-transfected with hK_Ca_2.2 and eGFP plasmid DNAs using standard lipofectamine (Thermo Fisher, USA) protocols. The cells were cultured in Dulbecco’s modified Eagle’s medium (DMEM1965, Thermo Fisher, USA) supplemented with 26.2 mM NaHCO_3_, 25 mM HEPES, 10 ml L^-1^ Glutamax (Gibco, USA), 10% fetal bovine serum (Biowest, France), and 100 U ml^-1^ of penicillin/streptomycin (Sigma, Germany); 1 to 2 days after the transfection, patch clamp experiments were conducted. On the day of the experiment, cells were detached from the flask using 1 ml of Detachin (Amsbio, United Kingdom) and plated on 0.5 mm Ø coverslips.

#### Cell Culture and Preparation for QPatch Experiments

The effect of ondansetron on I_KCa_ was studied in HEK293 cells stably expressing either hK_Ca_2.2 or hK_Ca_2.3 obtained from NeuroSearch A/S. The cell lines were established as described previously (Strøbaek et al., 2004). The cells were cultured in Dulbecco’s modified Eagle’s medium (DMEM1965, Thermo Fisher, USA) supplemented with 26.2 mM NaHCO_3_, 25 mM HEPES, 10 ml L^-1^ Glutamax (Gibco, USA), 10% fetal bovine serum (Biowest, France), 100 U ml^-1^ of penicillin/streptomycin (Sigma, Germany), and 100 µg/ml Geneticin (Gibco, UK)

The effect of AP14145 on K_v_11.1 has previously been described (Diness et al., 2017). The same method has been applied to study the effect of dofetilide and ondansetron using a stable CHO-K1 cell line expressing Kv11.1 on the automated QPatch machine. The cells were cultured in DMEM F12 (Sigma, Germany), 10% fetal bovine serum (Biowest, France) and 100 U ml^-1^ of penicillin/streptomycin (Sigma, Germany), 100 µg/ml Geneticin (Gibco, UK), 100 µg/ml hygromycin (Sigma-Aldrich), and 2 mM glutamine (Gibco, UK).

On the day of experiment, when cells reached about 70–90% confluency, they were detached with Detachin (Genlantis, USA) and resuspended in serum free medium (C5467 SAFC, Switzerland) containing 25 mM HEPES, 0.04 mg/ml soy bean trypsin inhibitor (T6522 Sigma-Aldrich), and 100 U/ml penicillin/streptomycin and were kept in suspension for QPatch experiments.

#### Recordings and Data Analysis

Manual patch clamp recordings were made using a HEKA EPC9 amplifier and the PatchMaster software (HEKA Elektronik, Germany) at room temperature using the whole cell configuration. Fluorescence was used to detect successfully transfected cells. Patch pipettes were pulled using a horizontal DMZ Universal Puller (Zeitz, Germany). I_KCa_ was elicited every 2 s using a 200-ms voltage ramp ranging −80 to +80 mV from a holding potential of 0 mV.

Automated patch-clamp whole-cell recordings were performed using a QPatch 16 HT system and disposable single-hole QPlates (Sophion, Denmark). Data were sampled at 10 kHz, four-order Bessel filter, cut-off frequency 3 kHz, and 80% Rs compensation.

I_KCa_ was elicited by a linear voltage ramp from –80 to +80 mV (200 ms in duration) applied every fifth second. The holding potential was 0 mV. Recordings were made before and after application of drug. The amount of inward current at –80 mV is used and plotted as a function of time to generate an I/t plot.

CHO cells expressing K_v_11.1 were held at –90 mV, and I_Kr_ was elicited by stepping to +20 mV for 2 s and then to –50 mV for 2 s. This second repolarizing step generates an outward tail current resulting from the recovery from inactivation. This tail current was plotted as function of time to generate an I/t plot.

For I_KCa_ and I_Kr_, the mean of the last three data points was used for further analysis. The currents were normalized using the last recordings prior to compound application as baseline and the current levels recorded in the presence of the positive control substances methyl-bicuculline for I_KCa_ or dofetilide for K_v_11.1 as reference for total inhibition of the channel. The normalized currents were then plotted as a function of drug concentration and fitted using the following equation on GraphPad Prism 7: Y = 100*(X^HillSlope)/(EC50^HillSlope + (X^HillSlope)), where Y is the normalized measured current, X is the logarithm of the dose of the tested drug, and IC_50_ the drug concentration needed to inhibit 50% of the current. Individual IC_50_ values were calculated for each experiment and then used to obtain the final mean ± S.E.M.

Correction for baseline drift was applied if applicable using the QPatch software.

### Quantitative Polymerase Chain Reaction

Five female Dunkin Hartley guinea pigs weighing 350–450 g were anesthetized and the hearts were quickly excised. The right atrium and left ventricle were separated and snap frozen in liquid nitrogen and kept at −80°C until RNA extraction.

Total RNA was extracted using the RNeasy Mini Kit (Qiagen, Manchester, UK) and was quantified using a NanoDrop spectrophotometer (NanoDrop, Thermo Scientific, Wilmington, USA). The RNA was reverse-transcribed using the nanoScript2 kit (Primerdesign Ltd., Southampton, UK). Negative controls (RT-) were made in the absence of the nanoScript2 enzyme and no template controls (NTCs) were run simultaneously with all reactions to assess contamination. All reactions were performed in duplicates using PrecisionPLUS 2xqPCR MasterMix (Primerdesign Ltd.) with Precision Bright White real-time PCR 96-well plates on the CFX Connect Real-Time System (Bio-Rad, Hertfordshire, UK). The cycling conditions were: initial activation at 95°C for 2 min, followed by 40 cycles of 95°C for 15 s, and 60°C for 1 min, and data were collected during each cycling phase. Cycle threshold (Ct) values were determined using Bio-Rad CFX96 Manager 3.0 software and the single threshold mode. Using a guinea pig geNorm Reference Gene Selection Kit (Primerdesign Ltd). and geNorm software the best reference genes and number of reference genes required for the most accurate gene normalization were determined (Vandesompele et al., 2002). The optimal number of reference genes was two (where geNorm V < 0.15): ACTB and SDHA. The relative expression of the genes of interest (*KCNN1*, *KCNN2*, *KCNN3*, and *KCNH2*) was calculated relative to the mean expression levels of the reference genes (*ACTB* and *SDHA*) using 2−ΔCt method (Livak and Schmittgen, 2001). All primers were double-dye (TaqMan style) and designed and optimized by Primerdesign Ltd. Primer sequences for guinea pig (Primerdesign Ltd.) are shown in **Supplementary Table 1**.

### Isolated Langendorff-Perfused Heart Preparations

The Langendorff perfused heart experiments were performed using a total of 24 female Dunkin Hartley guinea pigs (350–450 g, Charles River, Saint-Germain-Nuelle, France). Guinea pigs were anesthetized with an i.p. injection of pentobarbital 200 mg/ml, lidocaine hydrochloride 20 mg/ml, (Glostrup Apotek, Denmark), dose 0.150 ml/100 g body weight.

A tracheotomy was performed and the guinea pigs were ventilated with 60 strokes*5 ml/min using a rodent ventilator (7025 Rodent ventilator, Ugo Basile, Italy). The hearts were excised and cannulated *in situ* through a small puncture of the aorta and connected to the Langendorff retrograde perfusion setup (Hugo Sachs Elektronik, Harvard Apparatus GmbH, March, Germany). The hearts were retrogradely perfused at a constant perfusion pressure of 60 mmHg at 37°C, pH 7.4, Krebs-Henseleit buffer (in mM: NaCl 120, NaHCO_3_ 25, KCl 4, MgSO_4_ 0.6, NaH_2_PO_4_ 0.6, CaCl_2_ 2.5, glucose 11) saturated with 95% O_2_ and 5% CO_2_. The aortic perfusion pressure was determined with an ISOTEC transducer (Hugo Sachs Elektronik) and the coronary flow was measured with an ultrasonic flow meter (Transonic Systems Inc., USA). Both were connected to an amplifier (Hugo Sachs Elektronik). The electrical activity was measured using volume conducted ECGs and by placing epicardial monophasic action potential electrodes (MAPs) on the atria and ventricles. The hearts were immersed into a temperature-controlled bath containing pH 7.4 Krebs-Henseleit buffer. Perfusion pressure, coronary flow, ECG, and MAP analogue signals sampled at a frequency of 2k/s and converted by a 16/30 data acquisition system from PowerLab systems (ADInstruments, Oxford, UK) and monitored using LabChart 7 software (ADInstruments).

A bipolar pacing electrode was placed on the right atrial appendage for epicardial pacing stimulation using square pulses of 2 ms durations at three times diastolic threshold at 300 beats per minute. During the entire experiment, the ECG was monitored and monophasic action potential electrodes were placed on each chamber of the heart. The hearts were then left to stabilize for 20 min at intrinsic heart rhythm. The right AERP was measured by 10 regular stimuli (S1) followed by a premature extra stimulus (S_2_) applied with 1-ms increments. The AERP was defined as the longest S1–S2 interval failing to elicit an action potential.

#### Study Design

**Figure 1:**
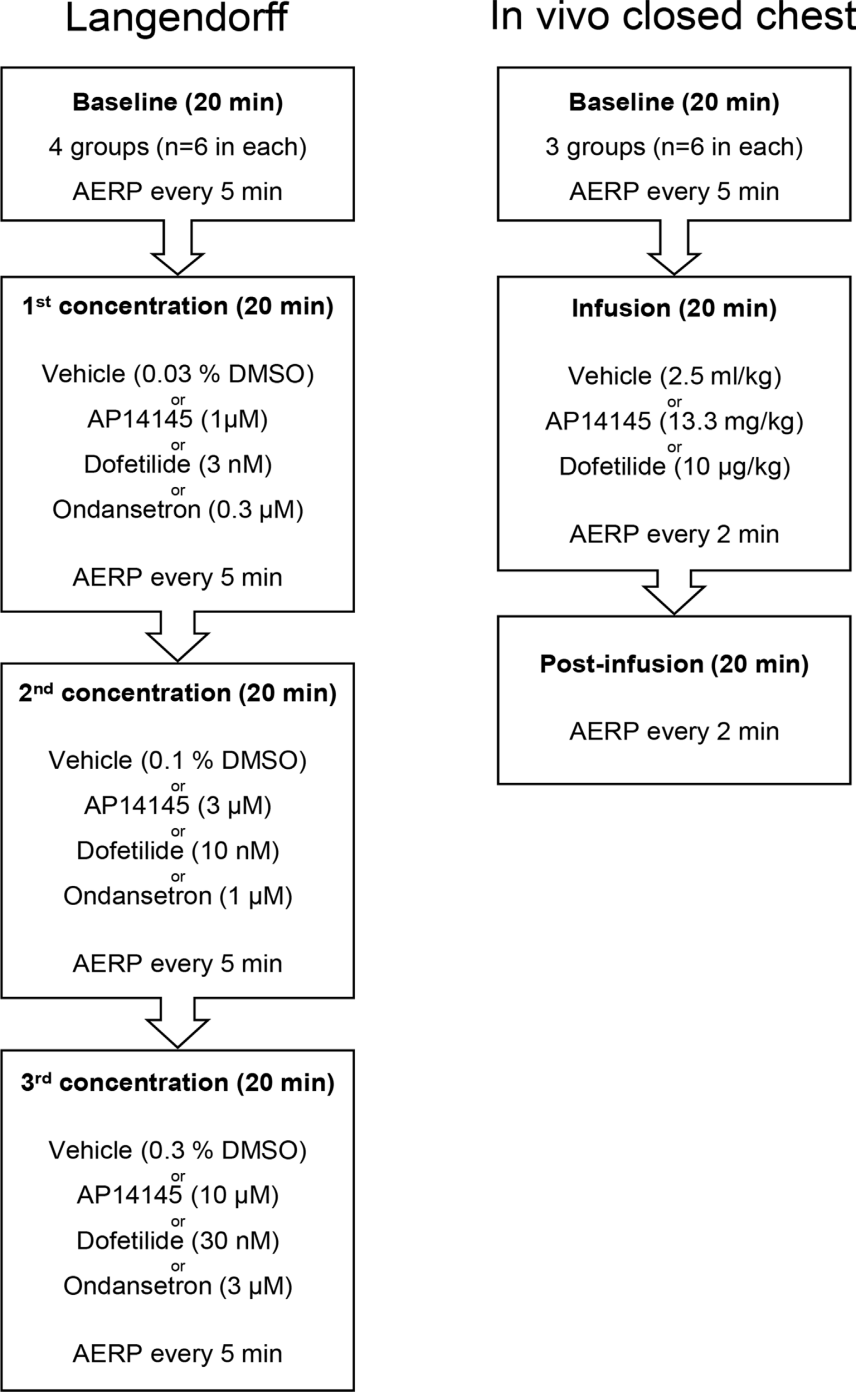
Flowchart of the Langendorff experiments (left) and *in vivo* closed chest recordings (right). See text for details.

Before each experiment isolated hearts were randomized to be perfused with AP14145, ondansetron, dofetilide, or vehicle (n = 6 for each group). After a period of stabilization, every Langendorff experiment consisted of four 20-min episodes (**Figure 1**). During the first 20-min episode, baseline parameters were recorded, and the following three 20-min episodes, the preparation was perfused with increasing concentrations of AP14145 (1, 10, and 30 µM), ondansetron (0.3, 1, and 3 µM) or dofetilide (3, 10, and 30 nM) or equivalent amounts of dimethylsulfoxide (DMSO) as the time matched control (TMC) group. During the entire experiment, the AERP was measured every 5 min and the ECG was continuously monitored.

### *In Vivo* Closed Chest Recordings

A total of 18 female guinea pigs (Dunkin Hartley HsdPoc : GH, Charles River) weighing 350–450 g were anesthetized with 5% isoflurane/oxygen in a sedation box. The guinea pigs were placed under artificial ventilation through a cannula placed in the trachea and anesthesia was reduced to 2.5% isoflurane/oxygen throughout the experiment. The temperature of the guinea pigs was monitored with a rectal thermometer and kept stable during the experiment using a heating lamp. Needle ECG electrodes were placed in each limb for ECG recordings (ADInstruments, UK). An electrophysiological catheter with eight electrodes (EPR-802) (Millar Inc., US) was placed in the right atrium *via* the right jugular vein. The two electrodes proximal to the tip of the catheter (Heeringa et al., 2006; Piccini et al., 2012) were used to pace the atrium and six electrodes (Waldo et al., 1996; Redfern et al., 2003; Roukoz and Saliba, 2007; Piccini et al., 2009; Grunnet et al., 2012; Zimetbaum, 2012) were used to measure the electrical activity in the atrium. The AERP was measured as in the Langendorff experiments. Between the AERP recordings, the heart remained unpaced. An intravenous catheter (inside diameter 0.5, outside diameter 0.8 mm, Natsume Seisakusho, Japan) was placed in the right jugular vein alongside the electrophysiology catheter for drug infusion.

#### Study Design

The animals were randomized to receive AP14145 (13.3 mg/kg, n = 6), dofetilide (0.01 mg/kg, n = 6), or vehicle (2.5 ml/kg, n = 6). First a period of 20 min baseline recording was conducted with AERP measurements every 5 min (**Figure 1**). After the baseline recording AP14145 (13.3 mg/kg), dofetilide (0.01 mg/kg), or vehicle was infused at a constant rate, over 20 min followed by a 20-min post-infusion period. The AERP was measured every 5 min during baseline and every 2 min during and after infusion.

### Data and Statistical Analysis

The data and statistical analysis complies with the recommendations on experimental design and analysis in pharmacology (Curtis et al., 2018). The operators in the *ex vivo* and *in vivo* experiments were not blinded to the treatment due to practical considerations. However, all data analysis was performed in a blinded fashion. ECG analyses were conducted in LabChart 7. Patch clamp recordings were acquired from PatchMaster and data analyses were performed using GraphPad Prism 7.0. Continuous data are summarized using the mean ± SEM. For the qPCR, two-tailed unpaired t-test was used to compare expression of the respective genes in atrium versus ventricle. Two-way ANOVA using Sidak’s *post hoc* test to compare differences in AERP, QTcB, and HR between the group receiving vehicle and groups receiving AP14145, ondansetron, or dofetilide. I_KCa_ was normalized to cell capacitance and paired one-way ANOVA with Dunnet’s *post hoc* test was performed to assess the effect of ondansetron and bicuculline methiodide. P values < 0.05 were considered significant and included in figures with three decimals.

## Results

### Expression of K_v_11.1 and K_Ca_2.x Channels in Guinea Pigs

**Figure 2:**
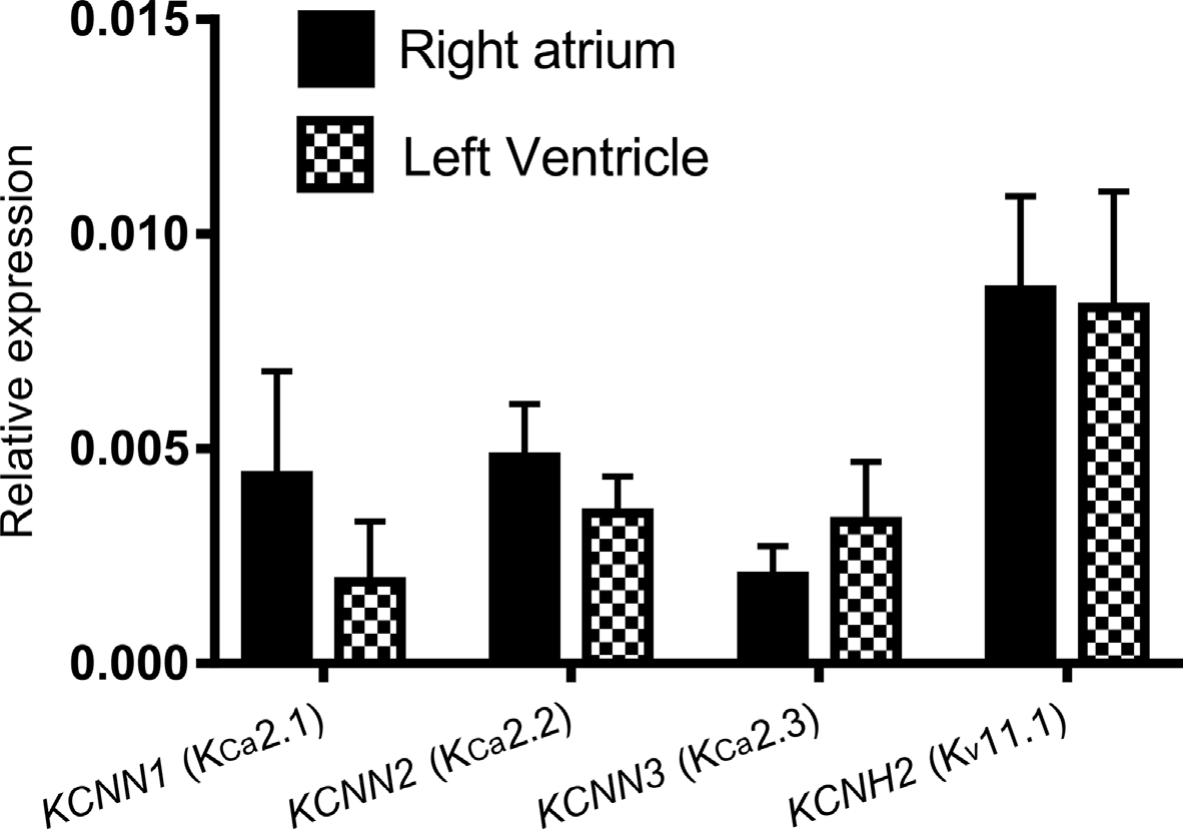
The expression of *KCNN1, KCNN2, KCNN3*, *and KCNH2* relative to the reference genes *ACTB* and *SDHA* using qPCR. No significant differences between the right atrium and the left ventricle are observed. Reactions were performed using tissue from five female Dunkin Hartley guinea pigs.

We performed qPCR to assess the expression levels of *KCNN1* (K_Ca_2.1), *KCNN2* (K_Ca_2.2), *KCNN3* (K_Ca_2.3), and *KCNH2* (K_v_11.1) mRNA in Dunkin Hartley guinea pigs. The guinea pigs showed a uniform expression of *KCNN1, KCNN2, KCNN3*, and *KCNH2* in atria and ventricles (**Figure 2**).

### In Vitro

A summary of IC_50_ values for ondansetron, dofetilide, and AP14145 on K_v_11.1 and K_Ca_2 channels published by our group previously or in the current study can be found in **Table 1**.

**Table 1:** Summary of IC_50_ values for ondansetron, dofetilide, and AP14145 on K_v_11.1 and K_Ca_2 channels published by our group previously or in the current study.

Ion channel	Method	AP14145 (IC_50_)	Dofetilide (IC_50_)	Ondansetron (IC_50_)
hK_Ca_2.2	Manual patch clamp	1.1 µM (Simó-Vicens et al., 2017b)		>1µM
hK_Ca_2.2	Automated patch clamp		60 µM (Simó-Vicens et al., 2017a)	>30 µM
hK_Ca_2.3	Manual patch clamp	1.1 µM (Simó-Vicens et al., 2017b)		
hK_Ca_2.3	Automated patch clamp	1.3 µM (Diness et al., 2017)	90 µM (Simó-Vicens et al., 2017a)	>30 µM
hK_v_11.1	Automated patch clamp	71.8 µM (Diness et al., 2017)	0.03 µM	2.79 µM

Values with no reference are from the present study.

#### Effect of Ondansetron on K_Ca_2 Channels

**Figure 3:**
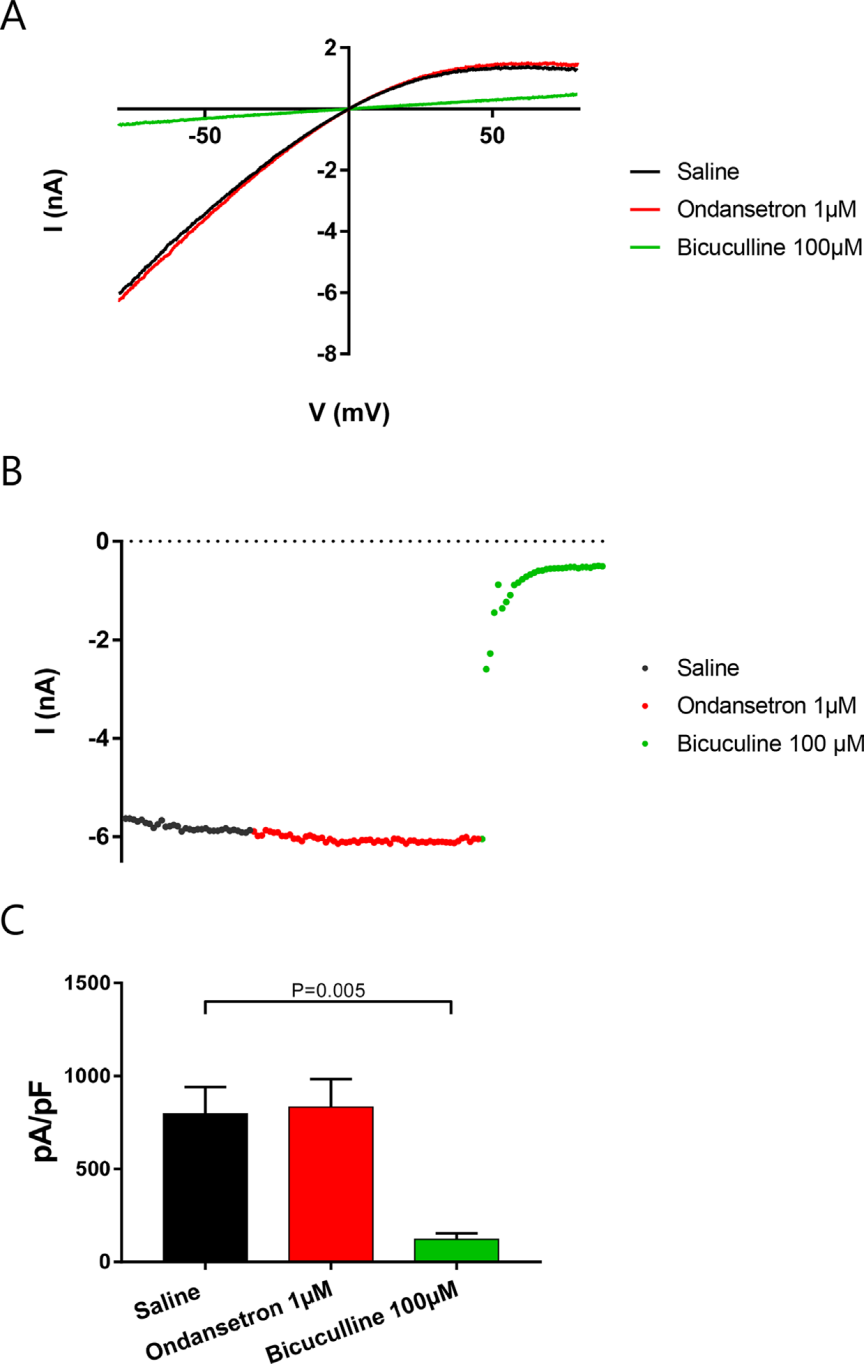
Effect of ondansetron on hK_Ca_2.2-expressing HEK cells showing that 1 µM of ondansetron did not inhibit I_KCa_. Voltage–current **(A)** and time–current **(B)** plots showing the effect of 1 µM ondansetron and 100 µM bicuculline methiodide. **(C)** A bar graph comparing the effects of ondansetron and bicuculline methiodide on I_KCa_.

Cells were dialyzed with 400 nM intracellular calcium and K_Ca_2.2 channels activated until I_KCa_ reached a steady state. Then, 1 µM ondansetron was applied for at least 2 min, followed by 100 µM bicuculline methiodide to inhibit the remaining current. Surprisingly, and in contrast to recent studies (Ko et al., 2018), ondansetron failed to inhibit I_KCa_ (n = 5, **Figure 3**).

**Figure 4:**
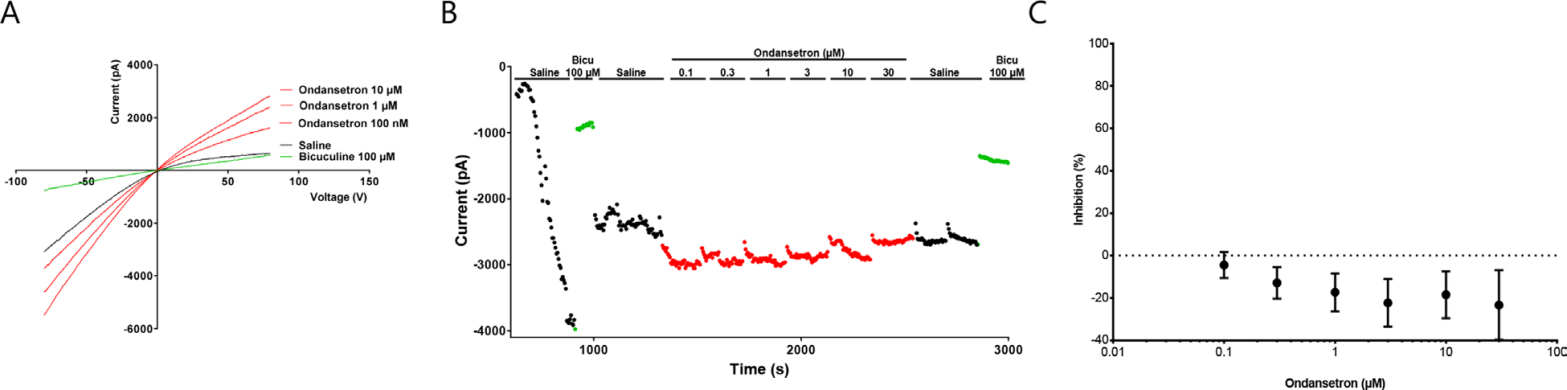
Effect of ondansetron on hK_Ca_2.3 current showing that up to 30 µM of ondansetron did not inhibit I_KCa_. Representative current trace **(A)** and current/time plot **(B)** showing the effect of increasing concentration of ondansetron. For visual clarity, only the effect of 100 nM, 1 µM, and 10 µM ondansetron is shown in the representative current trace. Currents for I/T plot were measured at the inward current at –80 mV (arrow). 100 µM of bicuculline was added as a reference drug at the beginning and end of the experiment. Because of a current run-up it seems like ondansetron has an activating effect; this run-up is compensated for in the I/T plot. **(C)** The inhibition curve shows that up to 30 µM ondansetron did not inhibit I_KCa_.

These findings were also confirmed by whole cell automated patch clamp recordings on stable cell lines expressing K_Ca_2.2 or K_Ca_2.3, when increasing concentration of ondansetron was added (in µM: 0.1, 0.3, 1, 3, 10, and 30) giving IC_50_ values of >30 µM for K_Ca_2.2 and K_Ca_2.3, respectively (n = 8, **Figure 4**).

#### I_Kr_ Is Inhibited by Dofetilide and Ondansetron But Not by AP14145

**Figure 5:**
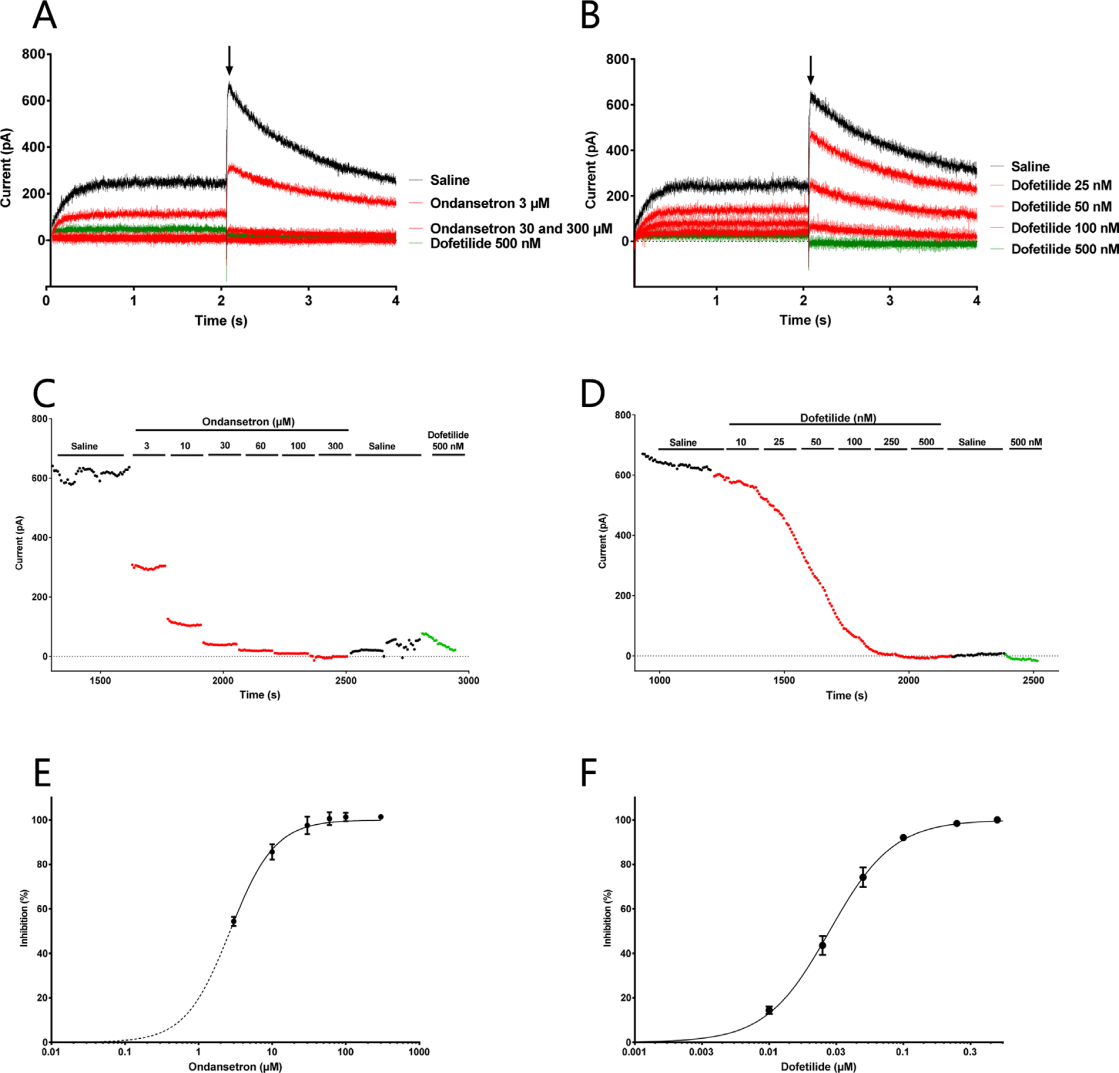
Effect of ondansetron (left) and dofetilide (right) on K_v_11.1 current showing that ondansetron blocks the I_Kr_ from 3 µM and dofetilide blocks from 0.03 µM. Representative current trace **(A, B)** and current/time plot **(C, D)** showing the effect of increasing concentration of ondansetron and dofetilide. For visual clarity, only the effects of 3, 30, and 300 µM ondansetron and 25, 50, and 100 nM dofetilide are shown in the representative current trace. Currents for the I/T plot are measured from the peak tail current (shown with an arrow). 500 nM dofetilide was added as a reference drug at the end of the experiment. **(E, F)** The inhibition curves showing that ondansetron and dofetilide both inhibit I_Kr_.

We have previously shown that the IC_50_ of AP14145 on K_v_11.1 was: 71.8 µM (Diness et al., 2017), and in this study we confirm that dofetilide, which is a known K_v_11.1 blocker, has an IC_50_ = 0.03 µM (n = 14) while ondansetron blocks K_v_11.1 with an IC_50_ = 2.79 ± 0.03 µM (n = 6, **Figure 5**).

### Effects on AERP, QTcB, and HR by AP14145, Ondansetron, and Dofetilide in Isolated Guinea Pig Hearts

**Figure 6:**
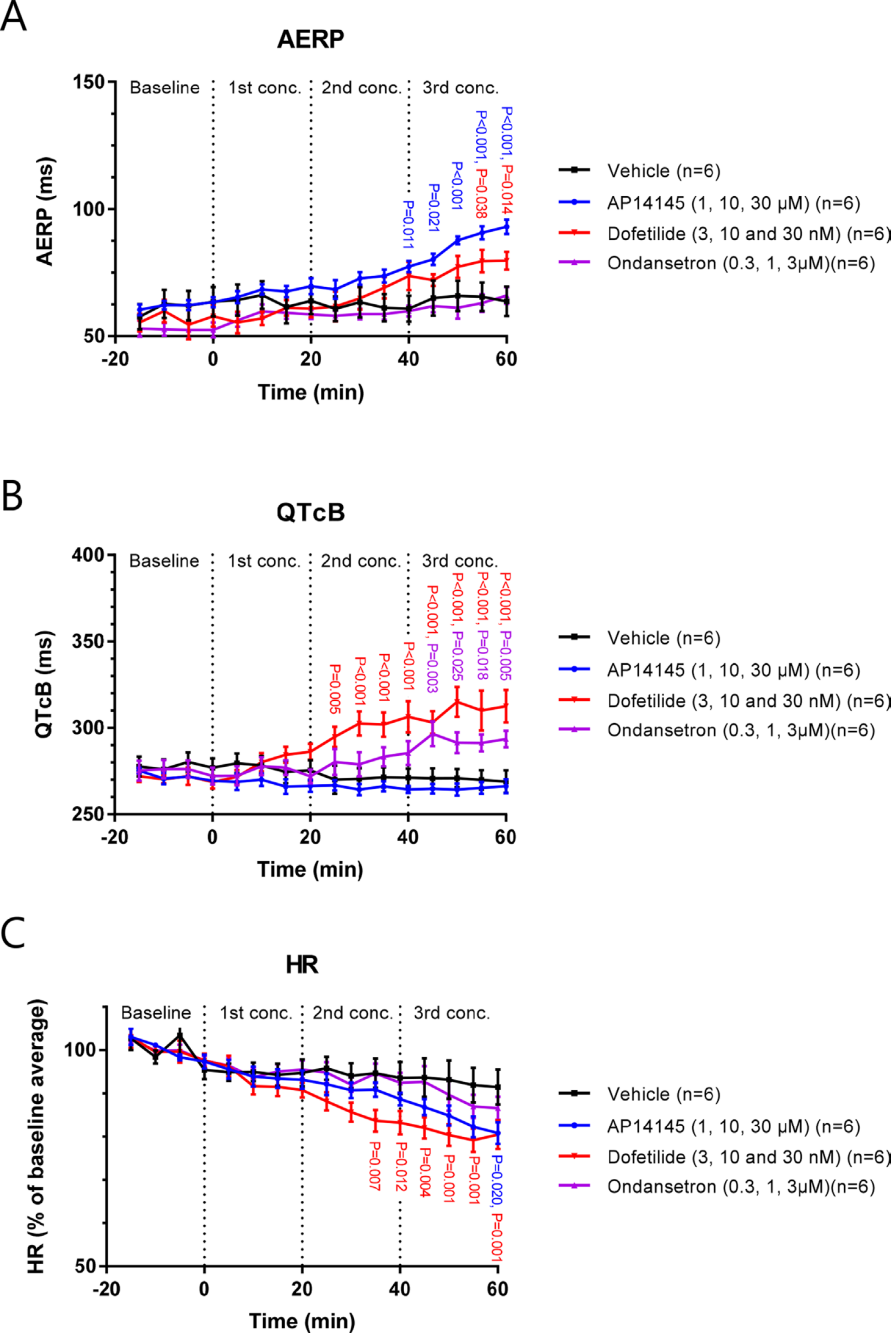
The effect of AP14145 (1, 10 and 30 µM), dofetilide (3, 10 and 30 nM), and ondansetron (0.3, 1 and 3 µM) on AERP **(A)**, QTcB **(B)**, and heart rate **(C)** was investigated in perfused Langendorff guinea pig hearts. P-values < 0.05 are given with three decimals in blue for AP14145, red for dofetilide, and purple for ondansetron.

In the Langendorff setup, AP14145 was added in increasing concentrations of 1, 10, and 30 µM to isolated guinea pig hearts. A significant AERP increase was observed at the end of wash-in with 10 µM compared to the vehicle TMC experiments (77 ± 2 ms vs. 61 ± 5 ms, P = 0.011). The AERP was further increased to a maximum of 93 ± 2 ms with 30 µM AP14145 compared to 64 ± 6 ms in the TMC group (P < 0.001). A concentration of 30 nM dofetilide increased AERP to 80 ± 4 ms compared to 64 ± 6 ms in the TMC group (P = 0.014). Ondansetron in concentrations up to 3 µM did not affect AERP significantly as compared to the TMC group (**Figure 6A**).

Dofetilide increased QTcB in concentrations of 10 and 30 nM to 306 ± 9 and 313 ± 9 ms, respectively, compared to TMC values of 271 ± 5 and 269 ± 6 ms (P < 0.001 in both cases). QTcB was also increased by 3 µM ondansetron to 294 ± 5 ms compared to the TMC value of 269 ± 6 ms (P = 0.005). AP14145 did not affect QTcB in any of the concentrations tested. At the highest concentration of AP14145 the QTcB was 266 ± 4 compared to the TMC values of 269 ± 6 ms (**Figure 6B**).

Dofetilide concentrations of 10 and 30 nM caused a decrease in HR to 83 ± 3% and 80 ± 3% of baseline values, which was significantly different from the TMC group where HR was decreased to 94 ± 4% and 91 ± 4% of baseline values at the corresponding time points (P = 0.012 and P < 0.001, respectively). HR was also decreased by 30 µM AP14145 to 81 ± 3% of the baseline value, which was significantly different from TMC (P = 0.020). Ondansetron in concentrations up to 3µM did not significantly affect HR when compared to the TMC group (**Figure 6C**).

### Effects on AERP, QTcB, and HR by AP14145 and Dofetilide *In Vivo*

**Figure 7:**
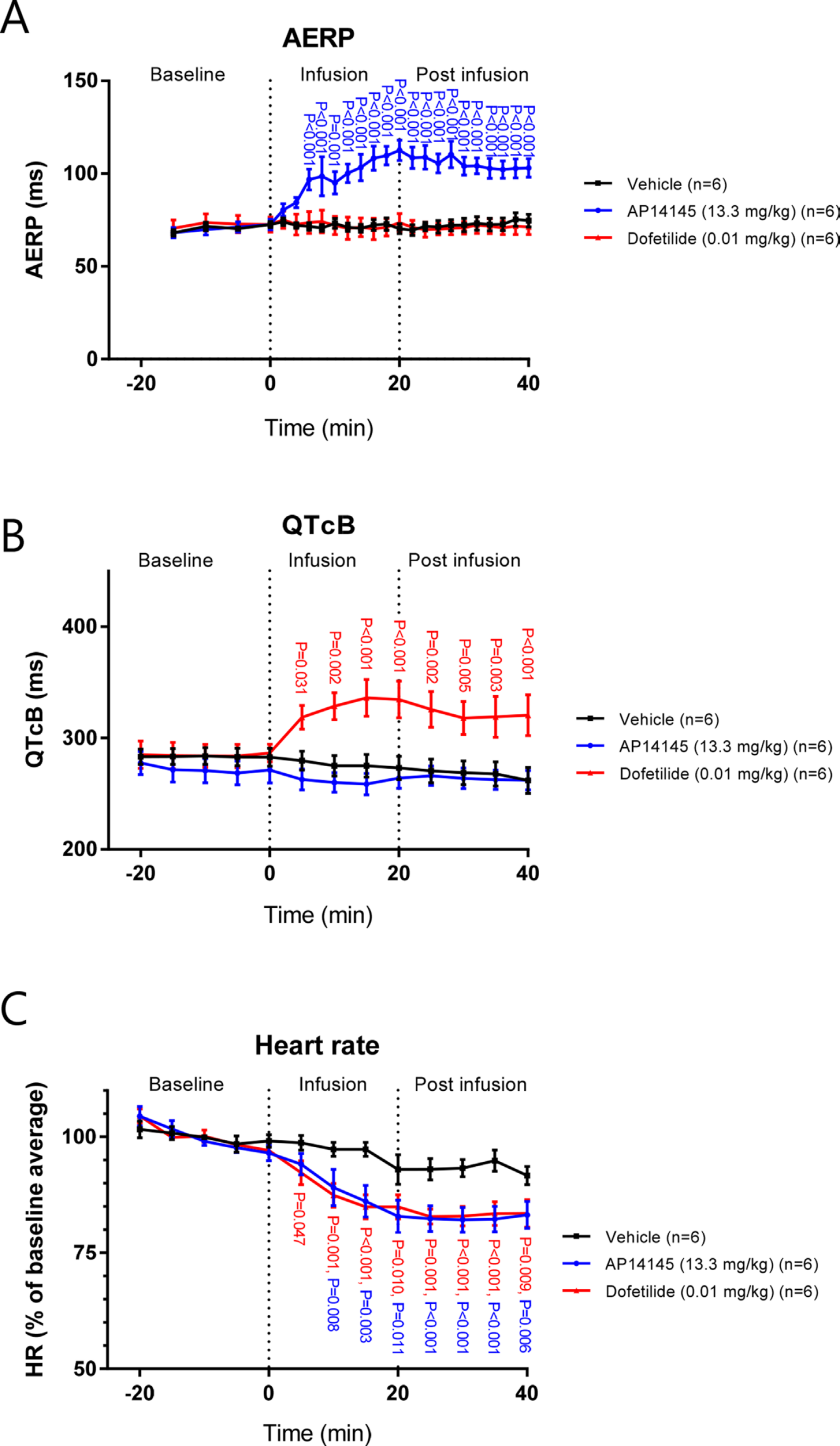
The effects of AP14145 (13.3 mg/kg) or dofetilide (0.01 mg/kg) on AERP **(A)**, QTcB **(B)**, and heart rate **(C)** were investigated in closed chest atrially paced guinea pigs. P-values < 0.05 are given with three decimals in blue for AP14145, and red for dofetilide.

In the *in vivo* guinea pig model, ECGs and atrial electrograms were recorded using needle electrodes and an intra-atrial catheter, respectively. After 6 min of infusion of AP14145 (at this time point 4 mg/kg had been given), the AERP was increased to 97 ± 6 ms compared to the TMC group in which AERP was 72 ± 2 ms (P < 0.001, **Figure 7A**).

AERP continually increased during the infusion of AP14145 to a maximum of 113 ± 6 ms compared to TMC (AERP = 75 ± 3 ms, P < 0.001). No effect on AERP of dofetilide infusion was observed.

As in the Langendorff experiments, no effect on QTcB with infusion of AP14145 was observed. In contrast, after 5 min of infusion the QTcB in the dofetilide group was increased to 319 ± 11 ms compared to the TMC group in which QTcB was 280 ± 9 ms (P < 0.031, **Figure 7B**). QTcB continually increased during the infusion of dofetilide to a maximum of 336 ± 16 ms compared to TMC (QTcB = 275 ± 10 ms, P < 0.001). During the infusion of AP14145 and dofetilide, the HR continuously declined to a minimum 5 min after the end of infusion. At this time point the HR in the AP14145 group and the dofetilide group was 83 ± 2% and 82 ± 3% of the baseline average and, respectively, which was in both cases lower than the HR of the TMC group, which was 93 ± 2% of the baseline average (P < 0.001, **Figure 7C**).

## Discussion

In this study it was compared how blocking two different K^+^ channels affects ventricular and atrial repolarization in guinea pigs. The effects of blocking the classical class III target, I_Kr_, were examined with the specific I_Kr_ blocker dofetilide. The effects of inhibiting the relatively new anti-AF target, I_KCa_, were examined with the tool compound AP14145. Since ondansetron is a known I_Kr_ blocker and was recently claimed to also be a potent blocker of I_KCa_, this compound was additionally examined in isolated hearts (Ko et al., 2018).

### Effects of the I_KCa_ Inhibitor AP14145

The I_KCa_ inhibitor, AP14145, prolonged AERP without increasing the QTcB interval, supporting the notion that I_KCa_ inhibition is a promising atrial specific antiarrhythmic target.

We hypothesized that this chamber difference was caused by differential expression of KCa2 channels in the atria vs ventricles. However, our qPCR data did not confirm this since no chamber specific distribution of *KCNN1* (K_Ca_2.1)*, KCNN2* (K_Ca_2.2), and *KCNN3* (K_Ca_2.3) were found on the transcriptional level. However, a number of posttranscriptional and posttranslational modifications may affect the actual protein levels. Furthermore, it can be speculated that differences between the precise subcellular location of the channels, action potential morphology, calcium dynamics, in atria and ventricles may affect activity of the channels and thus explain why I_KCa_ plays a more crucial role in the atria than in guinea pig ventricles under physiological conditions.

The HR was decreased by AP14145 both *ex vivo* and *in vivo*, which is in line with previous findings that I_KCa_ inhibition decreases sinoatrial activity (Chen et al., 2013; Torrente et al., 2017).

### Effects of the I_Kr_ Inhibitor Dofetilide

The I_Kr_ inhibitor dofetilide increased both ventricular and atrial repolarization in the isolated heart preparation. More pronounced I_Ks_ and I_Kr_ currents have been reported in the atria as compared to ventricles of guinea pigs (Sanguinetti and Jurkiewicz, 1991). However, surprisingly, ventricular repolarization was affected at lower concentrations of dofetilide than atrial repolarization. *In vivo*, dofetilide significantly prolonged the QTcB interval, but did not affect the AERP. Our functional data therefore suggest a more critical functional role of I_Kr_ in the ventricles compared to the atria in the guinea pig, which is comparable to what is seen in humans, where K_v_11.1 is more expressed in ventricles as compared to atria (Pond et al., 2000). The apparent functional ventricular selectivity of I_Kr_ inhibition cannot be explained by the expression data as we did not find any chamber specific distribution of *KCNH2* (K_v_11.1) on guinea pig mRNA level using quantitative PCR. Again, the explanation for this could be found among posttranscriptional and posttranslational factor affecting the actual protein levels as well as differences in the action potential morphology, which will lead to different activation of the K_v_11.1 channel in atria compared to ventricles.

The HR was decreased by dofetilide both *ex vivo* and *in vivo* in line with previous findings (Ruppert et al., 2016).

### Effects of Ondansetron

Ondansetron was included in this study since it has been reported to block both I_Kr_ and I_KCa_ at nanomolar concentrations (Kuryshev et al., 2000; Ko et al., 2018). Ondansetron is known to prolong the QT interval in patients (Benedict et al., 1996; Boike et al., 1997). This has traditionally been ascribed to its I_Kr_ blocking properties (Kuryshev et al., 2000). The data in the present study do lend support to the notion that ondansetron affects cardiac repolarization by inhibiting I_Kr_, but not by inhibiting I_KCa_. *In vitro* the compound did not affect HEK cells expressing K_Ca_2.2 channels (IC_50_ > 30 µM n = 8), nor K_Ca_2.3 channels (IC_50_ > 30 µM n = 8) but it did inhibit I_Kr_ in CHO-K1 cells expressing K_v_11.1 (IC_50_ = 2.79 ± 0.03 µM n = 6). Furthermore, in the isolated guinea pig hearts, the profile of ondansetron was similar to dofetilide and not to AP14145.

K_Ca_2 channels are widely expressed in the CNS and I_KCa_ inhibition is known to show neurological side effects in form of tremors when tested in conscious animals, probably due to the compound crossing the blood–brain barrier and blocking the neuronal K_Ca_2 channels (Stocker and Pedarzani, 2000; Simó-Vicens et al., 2017a). Avoiding CNS mediated adverse effects when developing compounds targeting peripheral K_Ca_2 channels would therefore be prudent. Indeed, changes in tremorography data are part of the primary tolerability/safety endpoints in the first clinical study with an I_KCa_ inhibitor, AP30663 (CHDR1706). If the CNS active compound ondansetron does indeed inhibit I_KCa_ it would be expected to cause tremors, which does not seem to be the case in patients treated for nausea and vomiting.

### Isolated Heart Preparation vs Closed Chest Preparation

AERP prolongation can translate to antiarrhythmic effect and is therefore a useful parameter in the development of antiarrhythmic compounds (Nattel, 2002; Comtois et al., 2005). The Langendorff model is a thoroughly validated model for measurements of AERP and QTcB. However, as the heart is excised from the body important neuro- and hormonal regulation of the heart is lost. This could potentially limit its usefulness as a tool for screening for novel antiarrhythmic drugs. In our experiments with AP14145 and dofetilide we found similar results for the Langendorff perfused heart experiments and closed chest recordings. This suggests that the isolated perfused heart preparation and the closed chest guinea pig model are equally suitable for evaluating AERP and QTcB effects of new chemical entities.

### Limitations

The sample size for most of the experiments in the current study is relatively small (n = 5 or 6) which increases the risk of making a type II error (wrongfully concluding that there is no difference between two samples). Thus, the study was only powered to detect relatively large and/or uniform differences between groups.

## Conclusion

In contrast to dofetilide and ondansetron, I_KCa_ inhibition by AP14145 increased AERP without prolonging QTcB in both Langendorff and a novel closed-chest guinea pig model. The data support that SK inhibition has the potential to be an effective approach in the treatment of AF.

Good consistency was observed between results obtained in isolated Langendorff hearts and in the novel *in vivo* guinea pig model.

## Ethics Statement

This study was performed under a license from the Danish Ministry of Justice (license no 2016-15-0201-00850) and in accordance with the recommendations of the Danish guidelines for animal experiments according to the European Commission Directive 86/609/EEC. The protocol was approved by the Danish Ministry of Justice.

## Author Contributions

JK, MS, KM, RS-V, SB, and LA performed the research. JD and BB designed the research study. US, MG, BB, and TJ contributed essential reagents or tools. JK, MS, RS-V, SB, and JD analyzed the data. JK and JD wrote the paper.

## Funding

This project has received funding from the European Union’s Horizon 2020 research and innovation program under the Marie Sklodowska-Curie grant agreement No. 675351 and the Innovation Fund Denmark.

## Conflict of Interest Statement

JD, US, MG, and BB are used by and have interests in Acesion Pharma and are inventors of Acesion Pharma patents within the field of SK channels.

The remaining authors declare that the research was conducted in the absence of any commercial or financial relationships that could be construed as a potential conflict of interest.
