# Supplementary material for: The KCa2 Channel Inhibitor AP14145, But Not Dofetilide or Ondansetron, Provides Functional Atrial Selectivity in Guinea Pig Hearts
Source: Front Pharmacol. 2019 Jun 19;10:668. doi: 10.3389/fphar.2019.00668 (PMC6593233; doi:10.3389/fphar.2019.00668)
Supplement: Supplementary file 1 [file Table_1.docx]

Supplementary table 1

Guinea pig qPCR primer sequences for *KCNN1*, *KCNN2*, *KCNN3* and *KCNH2.*

| Gene | Protein | Accession no. | Sense (s)  Anti-sense (as) | Sequence |
| --- | --- | --- | --- | --- |
| KCNN1 | SK1 | XM_013147404 | s | gaagcggctcagcgactat |
| KCNN1 | SK1 | XM_013147404 | as | ggtgtagacactccaggacag |
| KCNN2 | SK2 | XM_013156196 | s | tgcttggaaatactggtgtgtg |
| KCNN2 | SK2 | XM_013156196 | as | tggtagtggaaggggtatagga |
| KCNN3 | SK3 | XM_013158997 | s | tcagtctatccaccatcatcctg |
| KCNN3 | SK3 | XM_013158997 | as | gctatccgccagtcatccg |
| KCNH2 | Kv11.1 | NM_00117297 | s | aacttccgcaccacctacg |
| KCNH2 | Kv11.1 | NM_00117297 | as | tcctcaagagccagagccaa |
